# Development and validation of a predictive model for severe white matter hyperintensity with obesity

**DOI:** 10.3389/fnagi.2024.1404756

**Published:** 2024-06-03

**Authors:** Fu Chen, Lin-hao Cao, Fei-yue Ma, Li-li Zeng, Ji-rong He

**Affiliations:** ^1^Department of Neurology, Ruijin Hospital Luwan Branch, Shanghai Jiao Tong University School of Medicine, Shanghai, China; ^2^Department of General Medicine, Yinhang Community Health Centre, Shanghai, China; ^3^Department of Neurology and Institute of Neurology, Ruijin Hospital, Shanghai Jiao Tong University School of Medicine, Shanghai, China

**Keywords:** white matter hyperintensity, obesity, prediction models, uric acid, complement C3, interleukin 8

## Abstract

**Purpose:**

The purpose of the present study was to identify predictors of severe white matter hyperintensity (WMH) with obesity (SWO), and to build a prediction model for screening obese people with severe WMH without Nuclear Magnetic Resonance Imaging (MRI) examination.

**Patients subjects and methods:**

From September 2020 to October 2021, 650 patients with WMH were recruited consecutively. The subjects were divided into two groups, SWO group and non-SWO group. Univariate and Logistic regression analysis were was applied to explore the potential predictors of SWO. The Youden index method was adopted to determine the best cut-off value in the establishment of the prediction model of SWO. Each parameter had two options, low and high. The score table of the prediction model and nomogram based on the logistic regression were constructed. Of the 650 subjects, 487 subjects (75%) were randomly assigned to the training group and 163 subjects (25%) to the validation group. By resampling the area under the curve (AUC) of the subject’s operating characteristics and calibration curves 1,000 times, nomogram performance was verified. A decision curve analysis (DCA) was used to evaluate the nomogram’s clinical usefulness. By resampling the area under the curve (AUC) of the subject’s operating characteristics and calibration curves 1,000 times, nomogram performance was verified. A decision curve analysis (DCA) was used to evaluate the nomogram’s clinical usefulness.

**Results:**

Logistic regression demonstrated that hypertension, uric acid (UA), complement 3 (C3) and Interleukin 8 (IL-8) were independent risk factors for SWO. Hypertension, UA, C3, IL-8, folic acid (FA), fasting C-peptide (FCP) and eosinophil could be used to predict the occurrence of SWO in the prediction models, with a good diagnostic performance, Areas Under Curves (AUC) of Total score was 0.823 (95% CI: 0.760–0.885, *p* < 0.001), sensitivity of 60.0%, specificity of 91.4%. In the development group, the nomogram’s AUC (C statistic) was 0.829 (95% CI: 0.760–0.899), while in the validation group, it was 0.835 (95% CI: 0.696, 0.975). In both the development and validation groups, the calibration curves following 1,000 bootstraps showed a satisfactory fit between the observed and predicted probabilities. DCA showed that the nomogram had great clinical utility.

**Conclusion:**

Hypertension, UA, C3, IL-8, FA, FCP and eosinophil models had the potential to predict the incidence of SWO. When the total score of the model exceeded 9 points, the risk of SWO would increase significantly, and the nomogram enabled visualization of the patient’s WMH risk. The application prospect of our models mainly lied in the convenient screening of SWO without MRI examination in order to detect SWO and control the WMH hazards early.

## 1 Introduction

White matter hyperintensity (WMH), also known as leukoaraiosis or white matter lesions, is structural alteration to the brain that affects the white matter tracts specializing in higher brain activities. It is characterized by destruction to the myelin sheaths of central nerve cells. On imaging, the lesion presents as speckled or patchy changes in the periventricular or subcortical white matter, with high signal changed on MRI-T2-weighted images and fluid-attenuated inversion recovery (FLAIR) sequences (Fazekas et al., 1993). The lesion affects the white matter bundle, which is dedicated to higher brain functions. Cognitive decline, dementia, balance disorder, urinary dysfunction, and mood disorders are some of the principal clinical manifestations of WMH (Jing et al., 2017). It also raises the risk of stroke and mortality (Debette and Markus, 2010). WMH seriously influences the quality of life and self-care ability of patients, causing great harm to the health of the population. Therefore, it is very important and meaningful to explore the pathogenesis of WMH and take interventions based on the pathogenesis to control the WMH hazard.

Of individuals aged 1–45 years, 25.94% develop WMH (Wang et al., 2019). Whereas more than 50% of adults began to develop WMH in their 40 s (Wen et al., 2009), and the incidence rose sharply with advancing age (Moroni et al., 2018). In the Helsinki Aging Brain Study, 65% of people aged 70–75 years suffered from WMH (Ylikoski et al., 1995), while in more large cohort studies, a staggering 90% or more of people aged 80–90 years suffered from WMH (Longstreth et al., 1996; Garde et al., 2000; Deleeuw et al., 2001). The high prevalence of WMH seriously affected patients’ capacity for self-care and social functioning, putting their health at risk and placing a heavy burden on society. In order to manage WMH and enhance public health, it was crucial to investigate and define the mechanisms that cause these lesions as well as to pinpoint targets for intervention based on these mechanisms.

The pathogenesis of WMH is not fully understood. Numerous studies have been published to investigate the potential risk factors and pathogenesis of WMH, including oxidative stress, hypoperfusion, endothelial dysfunction, and blood–brain barrier impairment (Teoh et al., 2009). It is now widely acknowledged that hypertension, especially systolic blood pressure, is a independent risk factor for WMH (Dufouil et al., 2001). In addition, higher WMH volume has been linked to diabetes (van Harten et al., 2007) and smoking (Gons et al., 2011; Power et al., 2015). Studies have reported inconsistent findings regarding the role of inflammatory and metabolic factors in the pathogenesis of WMH. According to Giwa et al. (2012) and Zhang et al. (2015) a number of inflammatory proteins and cytokines, like complement C3 and C-reactive protein, could lead to cerebral microangiopathy and WMH through a series of reactions. Altendahl et al. (2020) and Boots et al. (2020) discovered that, however, a variety of variables, such as C-reactive protein and interleukins, were not substantially linked to WMH. WMH could be caused by various factors. Not all types of WMH were associated with inflammatory factors, which might be the reason for inconsistent conclusions. In addition, there might be different from WMH assessment methods, the type of institution where the patient was kept (general hospital, specialized hospital, rehab hospital, community hospital, research center, etc.), the main age groups of patients, and other varying factors in different research projects. These aspects could all produce erratic outcomes.

An estimated 1.9 billion individuals worldwide are either fat or overweight (Saltiel and Olefsky, 2017). Obesity raises disease mortality and increases the risk of a number of major illnesses, including cardiovascular diseases, type 2 diabetes, and tumors (Heron, 2018). Nowadays, it is widely acknowledged that obese patients suffer from chronic low inflammation. Such inflammation is also known as steatitis. Numerous inflammatory blood markers, including C-reactive protein, IL-6 and TNF are higher in these individuals (Rodríguez-Hernández et al., 2013; Dhananjayan et al., 2016). The anatomical and functional alterations in the brain as well as cognitive problems have been linked to this low-grade systemic inflammation (Haroon et al., 2012; Han et al., 2021). Obese patients have defective fibrinolytic systems, which might harm the endothelium of blood vessels. Additionally, obesity is linked to abnormal islet function, insulin resistance, and overexpression of monocyte chemotactic protein 1 (MCP-1) (Kahn and Flier, 2000; Parimisetty et al., 2016), which may affect energy homeostasis and cause excessive fat to accumulate in the liver, kidneys, heart, and other vital organs of the body, resulting in metabolic syndrome and increased organ load (Krzysztoszek et al., 2015).

Although there are no clear data on the rate of overlap between WMH and obesity, some studies had shown that people with high BMI were more likely to develop WMH (Lv et al., 2024). WMH and obesity were both associated with endothelial impairment and increased vascular load. Inflammatory and metabolic factors might have a more significant role in SWO. Therefore, a high rate of overlap between the two can be predicted. There was currently a lack of international research on SWO, so there was no research data to support the idea that there was a large number of patients with SWO, but as more relevant research is carried out in the future, more and more patients with SWO will be identified. The exploration of the pathogenesis of SWO, the screening, prediction, and intervention of SWO were also important for the health protection of the population.

The risk of illness beginning could be calculated using several indicators in prediction models, which could give a clearer, more understandable picture of the risk (Collins et al., 2015; Grant et al., 2018). In order to create a prediction model based on logistic regression coefficients that might more accurately predict the occurrence of SWO, we looked at various data on SWO, obesity, and WMH independently.

The aim of the present study is to identify predictors of SWO and to develop a prediction model to lay the foundation for further early diagnosis of the disease and early intervention, thereby reducing the hazard of WMH and improving the health of the WMH population.

## 2 Subjects and methods

### 2.1 Study subjects

**Figure 1:**
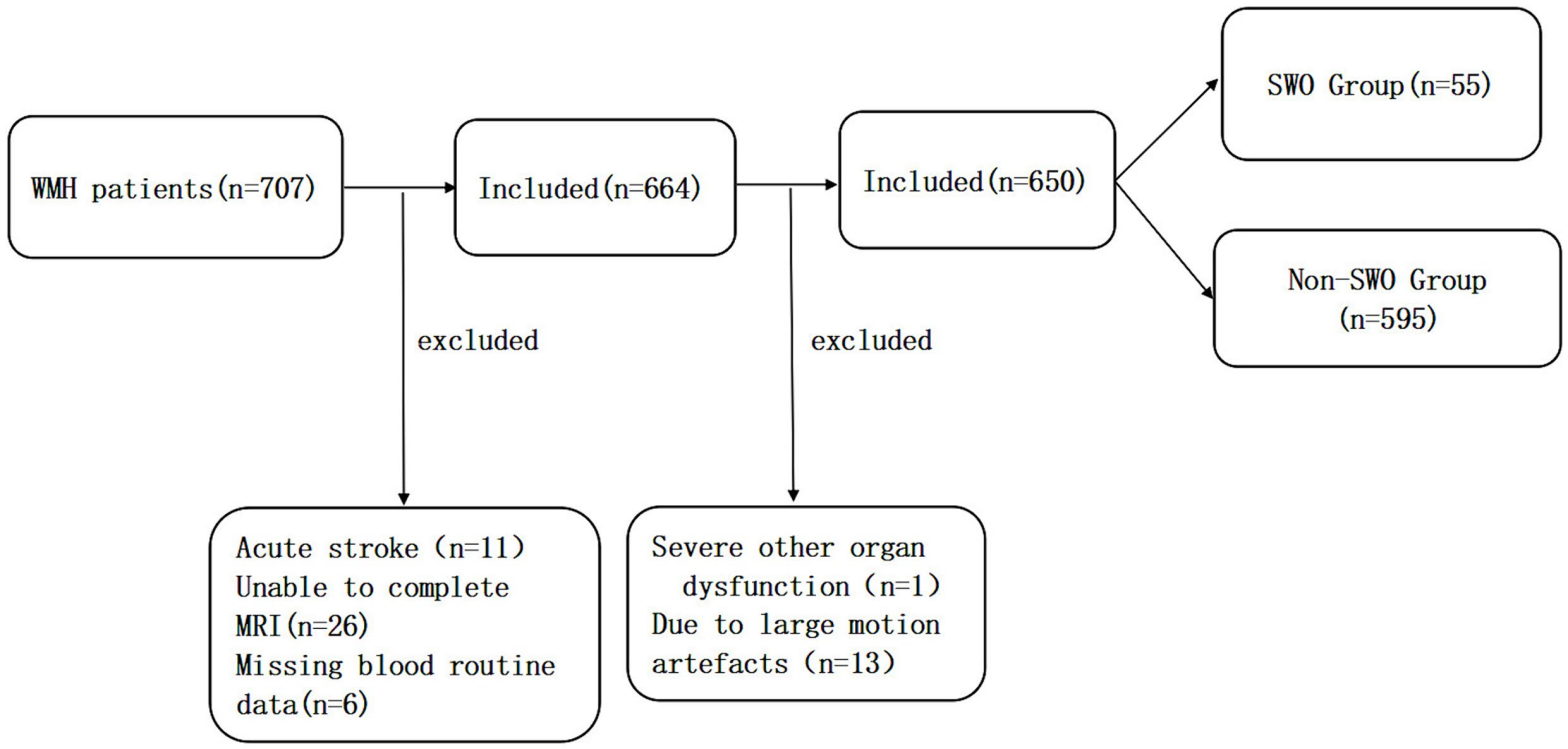
The recruitment of the subjects in this study.

From September 2020 to October 2021, 707 patients were screened consecutively in the Department of Neurology, Shanghai Jiaotong University School of Medicine affiliated Ruijin Hospital Luwan Branch. Inclusion criteria: patients over 18 years old who voluntarily participated in the study and signed informed consent. Exclusion criteria: (a) patients with acute disease conditions, such as acute infection, acute stroke, acute trauma and acute organ failure; (b) Patients who could not complete MRI. The study was approved by the Ethics Committee of Luwan Branch of Ruijin Hospital, Shanghai Jiao Tong University School of Medicine. All participants signed a written consent form. Among them, 57 patients did not meet the inclusion criteria, 11 patients had acute strokes, 26 patients were unable to complete MRI, 6 patients had no routine blood data, 1 patient suffered from severe other organ dysfunction and 13 patients had large motion artefacts on MRI. Eventually 650 people were recruited as study subjects (Figure 1).

### 2.2 Assessment of WMH

The head MRI (1.5 Tesla) plain scan was completed after admission. WMH was rated in accordance with the Fazekas visual rating scale (Fazekas et al., 1993). According to the method of Fazekas, the WMH was divided into periventricular white matter hyperintensity (PVWMH) and deep white matter hyperintensity (DWMH). The scoring criteria of PVWMH was as follows: grade 0: no WMH; grade 1: caps or pencil-thin lining; grade 2: smooth halo; grade 3: irregular PVWMH extending into the deep white matter. The DWMH scoring standard: grade 0: no WMHs; grade 1: punctate foci; grade 2: beginning confluence of foci; grade 3: large confluent areas. Total score: added the regional scores of the two to get the total score. All MRI evaluations were independently conducted by one experienced neurologist who was unaware of the other clinical conditions. Fazekas scores of 0 to 3 are considered mild WMH or no WMH, whereas scores of 4 to 6 are considered severe WMH.

### 2.3 Assessment of obesity

The most frequently utilized indicator of obesity in today is BMI due to its simplicity and viability. In this study, we measured obesity using BMI. For the measurements, participants wore light clothing, were barefoot or in stocking feet. BMI is determined by dividing weight (in kilograms) by square height (in meters). In China, adults with a BMI between 24 kg/m^2^ and 27.9 kg/m^2^ are defined as overweight and those with a BMI ≥ 28 kg/m^2^ are defined as obese (Zhou and Cooperative Meta-Analysis Group of the Working Group on Obesity in China, 2002; Zeng et al., 2021).

### 2.4 Clinical data collection

The clinical data of the study subjects was registered, including age, sex, height, weight, smoking and basic diseases such as hypertension, diabetes, hyperlipidemia, cardiac disease and previous strokes. The second day after admission, the fasting elbow vein blood was collected to complete the blood laboratory tests, including white blood cell (WBC), neutrophil, lymphocyte, monocyte, basophil, eosinophil, alanine aminotransferase (ALT), aspartate aminotransferase (AST), albumin, urea nitrogen (BUN), creatinine (Cr), uric acid (UA), complement, total cholesterol (TC), triglyceride (TG), high-density lipoprotein (HDL), low-density lipoprotein (LDL), free Interleukin 2 (Free IL-2), Interleukin β (IL-β), Interleukin 6 (IL-6), Interleukin 8 (IL-8), Interleukin 10 (IL-10), tumor necrosis factor-α (TNF-α), folic acid (FA), Vitamin B12 (VitB12), glycated hemoglobin (HbA1c), Immunoglobulin A (IgA), Immunoglobulin G (IgG), Immunoglobulin M (IgM), complement 3 (C3), complement 4 (C4), C-reactive protein (CRP), 25OH-Vitamin D (25OH-VitD), fasting blood glucose (FBG), 2-h postprandial blood glucose (2hPBG), fasting C-peptide (FCP), 2-h postprandial C-peptide (2hPCP).

### 2.5 Statistical analysis

SPSS 22.0 statistical software (IBM Corporation, Armonk, NY, United States) was adopted to process and analyze the data. The Kolmogorov Smirnov test was used to test the normality of the data. For the measurement data of normal distribution, the mean ± standard deviation (x ± s) was used to express, and the *t*-test of two independent samples was used to analyze. For the measurement data with non-normal distribution, it was expressed as the median (quartile) [M (P25, p75)], and Mann Whitney U rank sum test was used to analyze. Count data was expressed as relative number composition ratio (%) or rate (%), and chi square test was used to analyze. The correlation between the test group and the control group was analyzed by multivariate logistic regression. Receiver operating characteristic curve (ROC) was drawn to analyze the diagnostic efficacy of the prediction models. The level of statistical significance was set at 0.05. Youden index was used to determine the best cutoff value. The nomogram’s predictive performance was assessed using calibration curves, a decision curve analysis (DCA), and a concordance index (C-index). By computing the net benefits at various threshold probabilities, a decision curve analysis (DCA) was used to assess the nomogram’s clinical value (Vickers et al., 2008). Internal validation was also carried out using the 1,000 bootstrap resample validation method in order to increase the model’s accuracy and stability. Using the previously described techniques, we conducted DCA, ROC, C-index, and calibration curve studies for testing validation. The nomogram was constructed using the Brms^ Package of R4.3.0 statistical software (R Foundation for Statistical Computing, Vienna, Austria).

## 3 Results

### 3.1 A study on severe WMH with obesity

#### 3.1.1 Data characteristics

We excluded 57 non-compliant patients and finally a total of 650 patients met the inclusion criteria, including 399 males and 251 females, with an age range of 18–100 years and an average age of 67 ± 12 years. The population of the present study was Han nationality from mainland China. Between the SWO group (Fazekas = 4, 5, 6 and BMI ≥ 28 kg/m^2^, *n* = 55) and the non-SWO group (Fazekas = 0, 1, 2, 3 or BMI<28 kg/m^2^, *n* = 595), there were no obvious differences in sex, age, diabetes, hyperlipidemia, cardiac disease, previous strokes, smoking, neutrophil, lymphocyte, monocyte, basophil, ALT, AST, Albumin, BUN, Cr, complement, TC, TG, HDL, LDL, Free IL-2, IL-β, TNF-α, IL-10, IL-8, IL-6, FA, VitB12, HbA1c, IgA, IgG, IgM, CRP, 25OH-VitD, FBG, 2hPBG, FINS, 2hPINS, 2hPCP (*p* > 0.05), while, there were significant differences in hypertension, WBC, eosinophil, UA, C3, C4 and FCP (*p* < 0.05, Table 1).

**Table 1:** General characteristic analysis of patients in the SWO Group and the Non-SWO Group.

Variables	Total	SWO Group (*n* = 55)	Non-SWO Group (*n* = 595)	*p* value	
Age (year)		67.1 ± 11.5	64.1 ± 12.6	67.5 ± 11.4	0.668
Sex (*n*, %)					0.349
Male		399 (61.4%)	37 (63.7%)	362 (60.8%)	
Female		251 (38.6%)	18 (32.7%)	233 (39.2%)	
Hypertension		426 (65.5%)	50 (90.9%)	376 (63.2%)	< 0.001^*^
Diabetes	203 (31.2%)	20 (36.4%)	183 (30.8%)	0.391	
Hyperlipidemia	227 (34.9%)	24 (43.6%)	203 (34.1%)	0.157	
Cardiac disease	89 (13.7%)	10 (18.2%)	79 (13.3%)	0.311	
Previous strokes	209 (32.2%)	19 (34.5%)	190 (31.9%)	0.691	
Smoking	123 (18.9%)	8 (14.5%)	115 (19.3%)	0.386	
WBC (10^9/L)	6.5 ± 1.9	6.5 ± 1.7	6.5 ± 2.0	0.016^*^	
Neutrophil (10^9/L)	4.0 ± 1.5	4.0 ± 1.3	4.0 ± 1.6	0.656	
Lymphocyte (10^9/L)	1.8 ± 0.7	1.8 ± 0.7	1.8 ± 0.7	0.295	
Monocyte (10^9/L)	0.5 ± 0.1	0.5 ± 0.1	0.5 ± 0.1	0.297	
Basophil (10^9/L)	0.2 (0.1, 0.3)	0.2 (0.1, 0.4)	0.2 (0.1, 0.3)	0.068	
Eosinophil (10^9/L)	0.1 (0.1, 0.2)	0.2 (0.1, 0.3)	0.2 (0.1, 0.2)	0.021^*^	
ALT (IU/L)	17.0 (12.0, 24.0)	18.0 (13.0, 29.8)	17.0 (12.0, 24.0)	0.206	
AST (IU/L)	20.0 (16.0, 24.0)	19.0 (16.0, 23.0)	20.0 (16.0, 24.0)	0.579	
Albumin (g/L)	37.8 ± 3.4	39.3 ± 3.3	37.6 ± 3.4	0.216	
BUN (mmol/L)	5.8 ± 1.6	5.6 ± 1.5	5.9 ± 1.6	0.580	
Cr (umol/L)	80.0 ± 21.9	79.3 ± 16.8	80.2 ± 22.4	0.080	
UA (umol/L)	360.1 ± 96.7	393.1 ± 114.9	357.5 ± 94.1	0.005^*^	
Complement (U/mL)	49.8 ± 11.1	49.3 ± 12.4	49.8 ± 11.1	0.194	
TC (mmol/L)	4.3 ± 1.0	4.1 ± 1.1	4.3 ± 1.0	0.745	
TG (mmol/L)	1.4 ± 0.9	1.7 ± 0.9	1.4 ± 0.9	0.334	
HDL (mmol/L)	1.1(0.9, 1.3)	1.1(0.9, 1.2)	1.1(0.9, 1.3)	0.648	
LDL (mmol/L)	2.7 ± 0.8	2.6 ± 0.8	2.8 ± 0.8	0.410	
Free IL-2 (pg/mL)	563.8 ± 222.4	559.9 ± 269.1	561.9 ± 216.7	0.285	
IL-β (pg/mL)	3.9 (2.2, 4.7)	3.8 (2.8, 4.9)	3.7 (2.2, 4.7)	0.919	
TNF-α (pg/mL)	21.9 ± 12.8	25.7 ± 13.5	21.4 ± 12.7	0.500	
IL-10 (pg/mL)	2.6 (2.0, 3.4)	2.8 (2.0, 3.4)	2.6 (2.0, 3.4)	0.525	
IL-8 (pg/mL)	73.4 (40.4, 155.5)	112.0 (47.8, 237.5)	71.8 (39.4, 148.3)	0.051	
IL-6 (pg/mL)	3.7 (1.7, 5.8)	3.2 (1.1, 5.5)	3.4 (1.7, 5.8)	0.854	
FA (ng/mL)	8.7 ± 5.0	8.0 ± 3.7	8.8 ± 5.1	0.056	
VitB12 (pg/mL)	307.0 (219.0, 465.5)	300.5 (203.8, 484.0)	299.5 (219.0, 464.0)	0.341	
HbA1c (%)	5.9 (5.6, 6.7)	6.2 (5.8, 7.1)	5.9 (5.6, 6.7)	0.136	
IgA (mg/dL)	362.6 ± 117.1	262.8 ± 96.1	262.8 ± 119.7	0.805	
IgG (mg/dL)	1171.9 ± 291.6	1161.6 ± 307.2	1171.6 ± 291.5	0.215	
IgM (mg/dL)	80.5 (58.0, 108.8)	78.5 (62.8, 158.5)	79.0 (57.3, 108.0)	0.259	
CRP (mg/dL)	0.2 (0.2, 0.5)	0.3 (0.2, 0.6)	0.2 (0.1, 0.5)	0.127	
C3 (mg/dL)	88.0 ± 17.3	96.6 ± 14.7	87.1 ± 17.4	<0.001^*^	
C4 (mg/dL)	21.7 ± 6.6	23.3 ± 5.8	21.6 ± 6.7	0.007^*^	
25OH-VitD(nmol/L)	51.9 ± 23.1	48.2 ± 19.5	50.6 ± 21.7	0.519	
FBG (mg/dL)	5.8 ± 2.1	5.4 ± 1.1	5.9 ± 2.1	0.792	
2hPBG (mg/dL)	9.8 ± 3.2	10.2 ± 3.2	9.7 ± 3.5	0.214	
FCP (mg/mL)	2.8 ± 1.3	3.4 ± 1.0	2.6 ± 1.3	<0.001^*^	
2hPCP (ng/mL)	8.3 (5.6, 12.2)	10.1 (7.0, 12.9)	8.1 (5.4, 12.0)	0.270	

There were significant differences in hypertension, WBC, eosinophil, UA, C3, C4 and FCP (*p* < 0.05). The data was expressed as means and standard deviation or median and interquartile range for continuous variables, and counts and percentages for categorical variables. *p* values were obtained from *t*-test, rank sum test and χ 2 test. WBC, white blood cell; ALT, alanine aminotransferase; AST, aspartate aminotransferase; BUN, urea nitrogen; Cr, creatinine; UA, uric acid; TC, total cholesterol; TG, triglyceride; HDL, high-density lipoprotein; LDL, low-density lipoprotein; Free IL-2, free Interleukin 2; IL-β, Interleukin β; IL-10, Interleukin 10; IL-8, Interleukin 8; IL-6, Interleukin 6; FA,folic acid; VitB12, Vitamin B12; TNF-α, tumor necrosis factor-α; HbA1c,glycated hemoglobin; CRP,C-reactive protein; 25OH-VitD, 25OH-Vitamin D; C3,Complement 3; C4, Complement 4; FBG, fasting blood glucose; 2hPBG, 2-h postprandial blood glucose; FCP, fasting C-peptide; 2hPCP, 2-h postprandial C-peptide.

#### 3.1.2 Logistic regression analysis

In the logistic regression analysis, we included the factors with *p* < 0.1 in the multivariate analysis, and the results revealed that hypertension, IL-8, UA and C3 were independent risk factors for SWO (OR = 4.785, 95% CI: 1.850–12.377, *p* = 0.001, OR = 2.360, 95% CI: 1.232–4.521, *p* = 0.010, OR = 2.474, 95% CI: 1.376–4.448, *p* = 0.002, and OR = 4.031, 95% CI: 2.010–8.081, *p* = 0.000, respectively, Table 2).

**Table 2:** Logistic regression analysis of the clinical predictors of SWO.

	B	S.E.	Wald	Sig.	Exp (B)	95% C.I.
Hypertension	1.566	0.485	10.425	0.001	4.785	1.850–12.377
IL-8	0.859	0.332	6.698	0.010	2.360	1.232–4.521
UA	0.906	0.299	9.156	0.002	2.474	1.376–4.448
C3	1.394	0.355	15.426	0.000	4.031	2.010–8.081
Constant	−6.817	0.816	69.739	0.000	0.001	

Hypertension, IL-8, UA and C3 were risk factors for SWO. IL-8, Interleukin 8; C3, Complement 3 UA, uric acid.

### 3.2 A study on obesity

#### 3.2.1 Data characteristics

In order to examine the effect of obesity and severe WMH on SWO, we analyzed the obesity and WMH groups, respectively. We divided obesity factors into two groups, obesity group (BMI < 28 kg/m^2^, *n* = 544) and non-obesity group (BMI ≥ 28 kg/m^2^, *n* = 106). There were significant differences in hypertension, albumin, UA, C3, FA, 2hPBG, FCP between the two groups (*p* < 0.05), and the other factors were not statistically significant (*p* > 0.05, Table 3).

**Table 3:** Baseline feature comparisons between the obesity group and the non-obesity group.

Variables	Obesity group (BMI ≥ 28 kg/m^2^)	Non-obesity group (BMI < 28 kg/m^2^)	*p* value
Age	64.1 ± 11.2	67.8 ± 11.4	0.161
Hypertension	93 (87.7%)	333 (61.2%)	<0.001^*^
Diabetes	41 (38.7%)	162 (29.8%)	0.070
Hyperlipidemia	45 (42.5%)	182 (33.5%)	0.075
Cardiac disease	15 (14.2%)	74 (13.6%)	0.881
Previous strokes	34 (32.1%)	175 (32.2%)	0.985
Smoking	18 (17.0%)	105 (19.3%)	0.577
WBC (10^9/L)	6.1 ± 1.7	6.6 ± 2.0	0.141
Neutrophil (10^9/L)	3.7 ± 1.2	4.1 ± 1.6	0.975
Lymphocyte(10^9/L)	1.8 ± 0.7	1.9 ± 0.7	0.278
Monocyte (10^9/L)	0.4 ± 0.1	0.5 ± 0.1	0.419
Basophil (10^9/L)	0.2 (0.1, 0.3)	0.2 (0.1, 0.2)	0.221
Eosinophil (10^9/L)	0.1 (0.1, 0.3)	0.1 (0.1, 0.2)	0.518
ALT (IU/L)	19.0 (13.0, 31.0)	17.0 (12.0, 23.0)	0.063
AST (IU/L)	19.0 (16.0, 25.0)	20 (16.0, 23.0)	0.780
Albumin (g/L)	39.6 ± 3.1	37.5 ± 3.4	0.007^*^
BUN (mmol/L)	5.7 ± 1.5	5.9 ± 1.6	0.782
Cr (umol/L)	78.0 ± 14.2	80.4 ± 23.1	0.303
UA (umol/L)	385.0 ± 101.3	355.3 ± 95.4	0.002^*^
Complement (U/mL)	50.2 ± 10.7	49.7 ± 11.3	0.191
TC (mmol/L)	4.0 ± 1.1	4.3 ± 1.0	0.490
TG (mmol/L)	1.6 ± 0.7	1.4 ± 0.9	0.187
HDL (mmol/L)	1.0 (0.9, 1.1)	1.1 (0.9, 1.3)	0.525
LDL (mmol/L)	2.6 ± 0.8	2.8 ± 0.8	0.980
Free IL-2 (pg/mL)	559.9 ± 251.7	564.6 ± 217.3	0.497
IL-β (pg/mL)	3.8 (3.0, 4.7)	3.7 (2.2, 4.7)	0.857
TNF-α (pg/mL)	23.6 ± 14.3	21.6 ± 12.5	0.542
IL-10 (pg/mL)	2.7 (2.1, 3.3)	2.6 (2.0, 3.5)	0.996
IL-8 (pg/mL)	101.0 (47.1, 233.0)	71.6 (39.0, 146.0)	0.218
IL-6 (pg/mL)	3.3 (1.3, 5.6)	3.4 (1.7, 5.8)	0.755
FA (ng/mL)	8.1 ± 4.4	8.9 ± 5.1	0.024^*^
VitB12 (pg/mL)	299.0 (230.0, 445.0)	300.0 (215.5, 467.5)	0.825
HbA1c (%)	6.2 (5.8, 6.9)	5.9 (5.6, 6.7)	0.133
IgA (mg/dL)	268.7 ± 117.4	261.4 ± 117.4	0.329
IgG (mg/dL)	1153.2 ± 285.4	1175.4 ± 293.6	0.336
IgM (mg/dL)	72.0 (53.0, 110.0)	80.0 (59.5, 108.5)	0.955
CRP (mg/dL)	0.3 (0.2, 0.4)	0.2 (0.1, 0.5)	0.533
C3 (mg/dL)	95.0 ± 12.8	86.7 ± 17.8	<0.001^*^
C4 (mg/dL)	23.1 ± 5.9	21.5 ± 6.7	0.082
25OH-VitD(nmol/L)	49.7 ± 21.2	52.3 ± 23.5	0.349
FBG (mg/dL)	5.5 ± 1.2	5.9 ± 2.2	0.703
2hPBG (mg/dL)	10.8 ± 3.3	9.7 ± 3.1	0.039^*^
FCP (mg/mL)	3.4 ± 1.0	2.6 ± 1.3	<0.001^*^
2hPCP (ng/mL)	9.9 (5.8, 12.9)	8.1 (5.5, 11.9)	0.708

There were significant differences in hypertension, albumin, UA, C3, FA, 2hPBG, FCP between the two groups (*p* < 0.05).

#### 3.2.2 Logistic regression analysis

The risk factors of obesity were further analyzed by logistic regression. The results showed that hypertension (OR = 7.272, 95% CI: 1.650–32.044, *p* = 0.009), albumin (OR = 1.147, 95% CI: 1.018–1.293, *p* = 0.024), fasting C-peptide (OR = 1.443, 95% CI: 1.066–1.955, *p* = 0.018) were three independent risk factors for obesity group (Table 4).

**Table 4:** Logistic regression analysis of the risk factors of obesity.

	B	S.E.	Wald	Sig.	Exp (B)	95% C.I.
Hypertension	1.984	0.757	6.875	0.009	7.272	1.650–32.044
Albumin	0.137	0.061	5.066	0.024	1.147	1.018–1.293
FCP	0.367	0.155	5.629	0.018	1.443	1.066–1.955
Constant	−9.684	2.574	14.159	0.000	0.000	

Hypertension, albumin and FCP were risk factors for obesity group. FCP, fasting C-peptide.

### 3.3 A study on WMH

#### 3.3.1 Data characteristics

Similarly, we divided the recruited subjects into two groups. One group of patients with Fazekas score greater than or equal to 4 was defined as severe WMH group (*n* = 289), and the other group of patients with Fazekas score less than or equal to 3 was defined as non- severe WMH group (*n* = 361). Between the two groups，there were statistically significant differences in age, hypertension, previous strokes, WBC, neutrophil, monocyte, eosinophil, albumin, BUN, Cr, free IL-2, IgA, IgG, CRP, C4 (*p* < 0.05), and the other factors were not significantly different (*p* > 0.05, Table 5).

**Table 5:** Baseline feature comparisons between the severe WMH Group and the non-severe WMH Group.

Variables	Severe WMH group (Fazekas = 4,5,6)	Non-severe WMH group (Fazekas = 0,1,2,3)	*p* value
Age	70.7 ± 10.3	63.8 ± 11.6	<0.001^*^
Hypertension	222 (76.8%)	204 (56.5%)	<0.001^*^
Diabetes	99 (34.3%)	104 (28.8%)	0.136
Hyperlipidemia	96 (33.2%)	131 (36.3%)	0.415
Cardiac disease	42 (14.5%)	47 (13.0%)	0.577
Previous strokes	110 (38.1%)	99 (27.4%)	0.004^*^
Smoking	58 (20.1%)	65 (18.0%)	0.504
WBC (10^9/L)	6.5 ± 1.9	6.5 ± 2.0	0.007^*^
Neutrophil (10^9/L)	4.0 ± 1.4	4.0 ± 1.6	0.026^*^
Lymphocyte(10^9/L)	1.8 ± 0.7	1.9 ± 0.6	0.188
Monocyte (10^9/L)	0.5 ± 0.1	0.4 ± 0.2	<0.001^*^
Basophil (10^9/L)	0.2 ± 0.1	0.2 ± 0.1	0.541
Eosinophil (10^9/L)	0.2 ± 0.1	0.2 ± 0.1	0.048^*^
ALT (IU/L)	16.0 (12.0, 23.0)	18.0 (13.0, 25.0)	0.792
AST (IU/L)	19.0 (16.0, 23.0)	20.0 (16.0, 24.0)	0.207
Albumin (g/L)	37.4 ± 3.5	38.3 ± 3.4	0.001^*^
BUN (mmol/L)	6.0 ± 1.6	5.6 ± 1.6	0.001^*^
Cr (umol/L)	83.3 ± 23.3	76.9 ± 20.1	0.001^*^
UA (umol/L)	368.9 ± 94.5	351.6 ± 98.6	0.122
Complement (U/mL)	49.0 ± 12.1	50.4 ± 10.1	0.390
TC (mmol/L)	4.2 ± 1.0	4.4 ± 1.1	0.953
TG (mmol/L)	1.4 ± 0.7	1.5 ± 1.0	0.226
HDL (mmol/L)	1.1 (0.9, 1.3)	1.0 (0.9, 1.3)	0.253
LDL (mmol/L)	2.7 ± 0.8	2.8 ± 0.8	0.890
Free IL-2 (pg/mL)	580.6 ± 244.7	547.6 ± 198.6	0.001^*^
IL-β (pg/mL)	3.6 (2.1, 4.8)	3.7 (2.6, 4.5)	0.476
TNF-α (pg/mL)	23.7 ± 12.7	20.2 ± 12.7	0.070
IL-10 (pg/mL)	2.5 (2.0, 3.4)	2.6 (2.0, 3.5)	0.629
IL-8 (pg/mL)	85.9 (52.178)	62.4 (27.4, 141.3)	0.648
IL-6 (pg/mL)	3.7 (1.6, 6.7)	3.2 (1.7, 5.3)	0.612
FA (ng/mL)	8.7 ± 4.7	8.7 ± 5.3	0.067
VitB12 (pg/mL)	295.0 (196.8, 484.0)	304.0 (231.0, 464.5)	0.315
HbA1c (%)	5.9 (5.7, 6.6)	5.9 (5.6, 6.8)	0.346
IgA (mg/dL)	272.2 ± 123.3	253.3 ± 110.7	0.004^*^
IgG (mg/dL)	1171.1 ± 282.1	1172.6 ± 302.1	0.007^*^
IgM (mg/dL)	79.0 (56.5, 111.3)	80.0 (58.0, 108.0)	0.430
CRP (mg/dL)	0.3 (0.2, 0.5)	0.2 (0.1, 0.4)	0.015^*^
C3 (mg/dL)	86.9 ± 17.3	89.2 ± 17.4	0.934
C4 (mg/dL)	22.0 ± 7.1	21.4 ± 6.2	0.005^*^
25OH-VitD (nmol/L)	51.1 ± 23.0	52.7 ± 23.3	0.511
FBG (mg/dL)	5.6 ± 1.6	6.1 ± 2.4	0.284
2hPBG (mg/dL)	9.7 ± 2.9	10.0 ± 3.4	0.522
FCP (mg/mL)	2.8 ± 1.1	2.7 ± 1.4	0.857
2hPCP (ng/mL)	8.5 (6.1, 12.2)	8.1 (5.0, 12.3)	0.210

There were statistically significant differences in age, hypertension, previous strokes, WBC, neutrophil, monocyte, eosinophil, albumin, BUN, Cr, free IL-2, IgA, IgG, CRP, C4 (*p* < 0.05).

#### 3.3.2 Logistic regression analysis

These factors were incorporated in logistic regression analysis, and the results demonstrated that age (OR = 1.058, 95% CI: 1.026–1.091, *p* = 0.000) and hypertension (OR = 2.738, 95% CI: 1.405–5.335, *p* = 0.003) were two independent risk factors for severe WMH group (Table 6). Hypertension was a specific risk factor for SWO.

**Table 6:** Logistic regression analysis of the risk factors for severe WMH.

	B	S.E.	Wald	Sig.	Exp (B)	95% C.I.
Age	0.056	0.016	12.831	0.000	1.058	1.026–1.091
Hypertension	1.007	0.340	8.756	0.003	2.738	1.405–5.335
Constant	−4.618	1.110	17.324	0.000	0.010	

Age and hypertension were risk factors for severe WMH group.

## 4 Prediction model and nomogram of factors associated with SWO

### 4.1 Prediction model for SWO

Of the 650 subjects, 487 subjects (75%) were randomly assigned to the training group and 163 subjects (25%) to the validation group. There was no statistical significance between the training and validation groups (Supplementary Table S1). We constructed ROC curves of SWO and found that the area under the curve (AUC) values of IL-8, UA and C3 were all >0.5, *p* < 0.05. The Youden index was used to determine the best cutoff value. Each parameter had two options, low and high, and each option of the parameter was corrected by a defined point. Based on the coefficients calculated by the logistic regression model, the HUCI score was created, including four variables: hypertension, UA, C3 and IL-8. In order to improve the efficiency of the prediction model, we tried to include the classification data of the factors with *p* values less than 0.1 into the model. The detailed cut-off values, sensitivity, specificity and diagnostic rate of each parameter were shown in Table 7.

**Table 7:** The determination of the AUC and the cutoff values of SWO risk factors by means of logistic regression, ROC curves, and the Youden index.

	AUC	*p*	Upper	Lower	Cutoff value	Sensitivity	Specificity
UA(umol/L)	0.622	0.003	0.538	0.707	402	0.509	0.741
IL8(pg/mL)	0.623	0.003	0.548	0.698	79.4	0.731	0.511
C3(mg/dL)	0.682	<0.001	0.613	0.751	91	0.800	0.520
C4(mg/dL)	0.603	0.011	0.520	0.686	26	0.491	0.741
FA(ng/mL)	0.562	0.130	0.479	0.645	13.8	0.327	0.836
Eosinophil(10^9/L)	0.638	0.001	0.564	0.713	0.24	0.400	0.807
FCP(mg/mL)	0.691	<0.001	0.623	0.760	2.5	0.836	0.530
WBC(10^9/L)	0.585	0.037	0.506	0.664	6.51	0.564	0.618

IL-8, Interleukin 8; C3, Complement 3; C4, Complement C4; UA, uric acid; FA, folic acid; FCP, fasting C-peptide; WBC, white blood cell.

Through logistic regression analysis, we created the HUCIFFE score, which includes seven variables: hypertension, UA, C3, IL-8, FA, FCP and eosinophil (Table 8).

**Table 8:** Logistic regression analysis of risk factors for the HUCIFFE model.

	B	S.E.	Z	Sig.	Exp (B)	95% C.I.
Hypertension	1.65	0.54	3.09	0.002	5.23	1.83 ~ 14.93
IL-8	0.86	0.36	2.43	0.015	2.37	1.18 ~ 4.77
C3	1.37	0.39	3.51	<0.001	3.92	1.83 ~ 8.41
UA	0.92	0.33	2.78	0.005	2.52	1.31 ~ 4.82
FCP	1.89	0.45	4.18	<0.001	6.61	2.73 ~ 16.04
FA	0.83	0.36	2.32	0.020	2.30	1.14 ~ 4.64
Eosinophil	1.00	0.34	2.92	0.003	2.72	1.39 ~ 5.31

IL-8, Interleukin 8; C3, Complement 3; UA, uric acid; FA, folic acid; FCP, fasting C-peptide.

**Figure 2:**
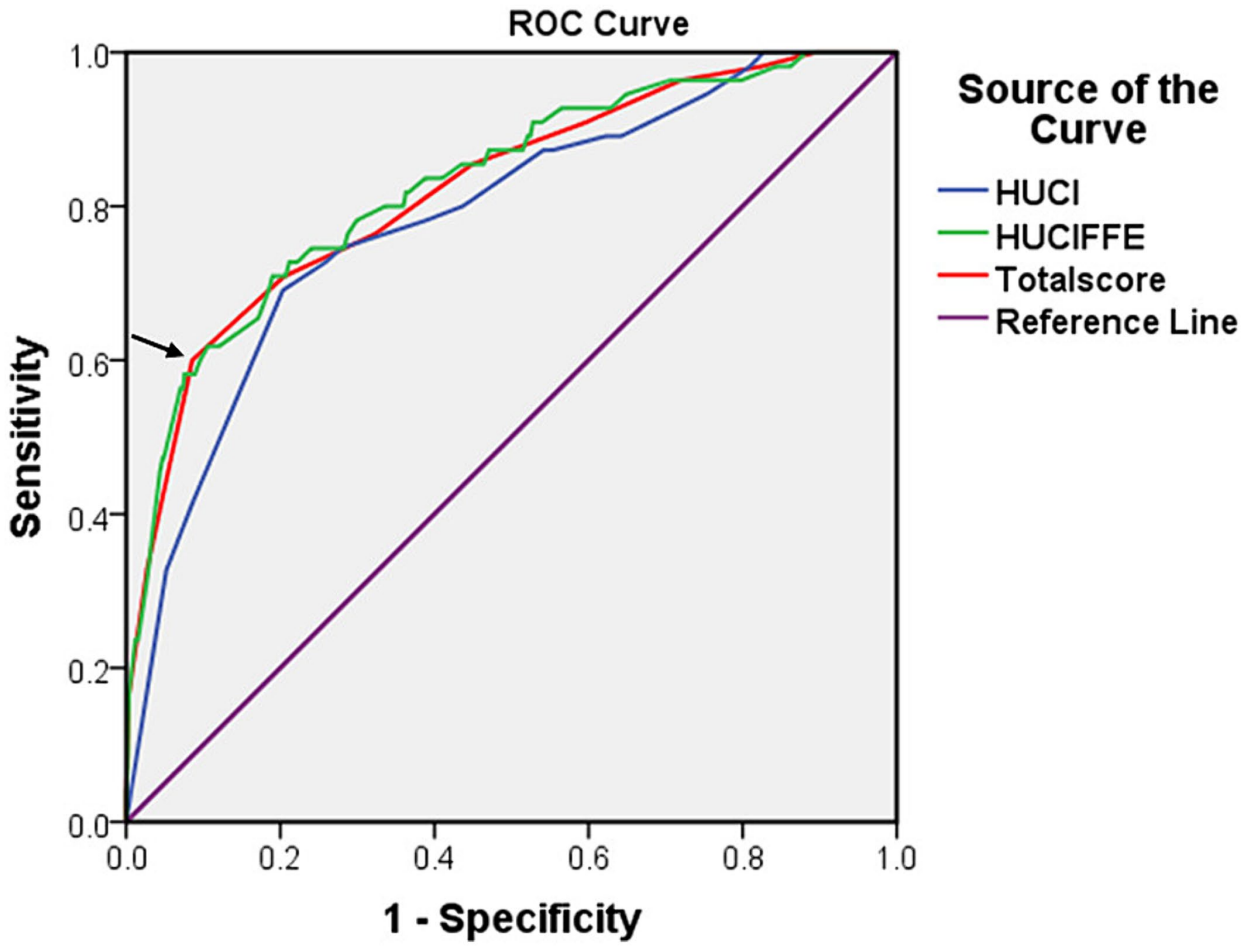
The receiver operating characteristic (ROC) curves for the HUCI score (hypertension, UA, C3 and IL-8), the HUCIFFE score (hypertension, UA, C3, IL-8, FA, FCP and eosinophil) and the Total score for 650 patients. The arrow represents the cut-off value.

The AUCs of HUCI score and HUCIFFE score were 0.785 (95% CI: 0.720–0.850, *p* < 0.001) and 0.829 (95% CI: 0.768–0.890, *p* < 0.001), respectively. The efficacy test of HUCIFFE score was better than that of HUCI score, and had good predictive performance. The sensitivity and specificity were 70.9 and 81.0%, respectively (Figure 2).

The receiver operating characteristic (ROC) curves for the HUCI score (hypertension, UA, C3 and IL-8), the HUCIFFE score (hypertension, UA, C3, IL-8, FA, FCP and eosinophil) and the Total score for 650 patients. The arrow represents the cut-off value.

We added the total scores of the seven classification factors, and used Youden index to determine the best cutoff value of the total score, then, we could judge the disease risk. Based on the coefficients calculated by the logistic regression model, we developed a scoring table for predicting SWO using the classification variables of hypertension, UA, C3, IL-8, FA, FCP and eosinophil. The AUCs of Total score was 0.823 (95% CI: 0.760–0.885, *p* < 0.001), respectively. The sensitivity and specificity were 60.0 and 91.4%, respectively (Figure 2; Table 9).

**Table 9:** SWO score system.

Item	Classification	Cutoff	Points
Hypertension	Yes No		3 0
IL-8 (pg/mL)	High Low	≥79.4 <79.4	1 0
C3 (mg/dL)	High Low	≥91 <91	2 0
UA (umol/L)	High Low	≥402 <402	1 0
FA (ng/mL)	High Low	≥13.8 <13.8	2 0
FCP (mg/mL)	High Low	≥2.5 <2.5	2 0
Eosinophil (10^9/L)	High Low	≥0.24 <0.24	1 0
Total score	High Low	≥9 <9	

IL-8, Interleukin 8; C3, Complement 3; UA, uric acid; FA, folic acid; FCP, fasting C-peptide. The risk factor scores for SWO were summed to create a total score, and when the total score was greater than 9, the risk of developing SWO was significantly higher.

### 4.2 Development and validation of predictive nomogram

#### 4.2.1 Model development

**Figure 3:**
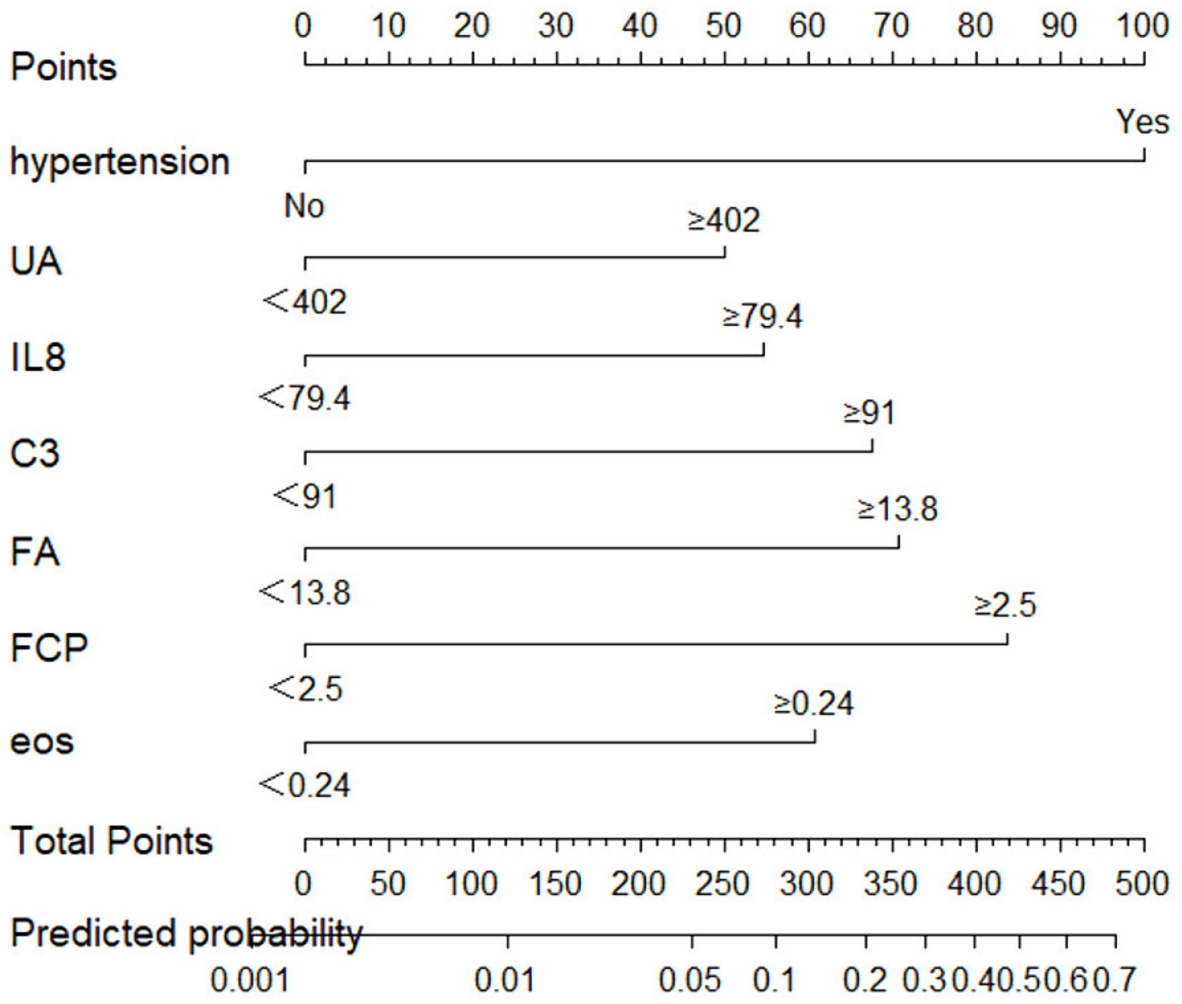
Nomogram for predicting SWO probabilities.

**Figure 4:**
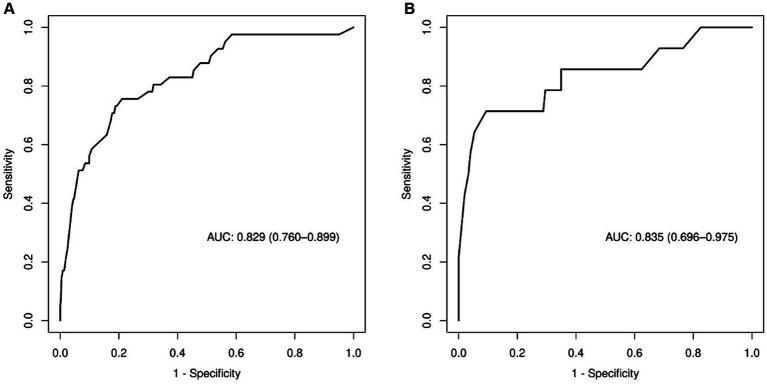
The (ROC) curve of the nomogram. **(A)** Training set; **(B)** validation set.

**Figure 5:**
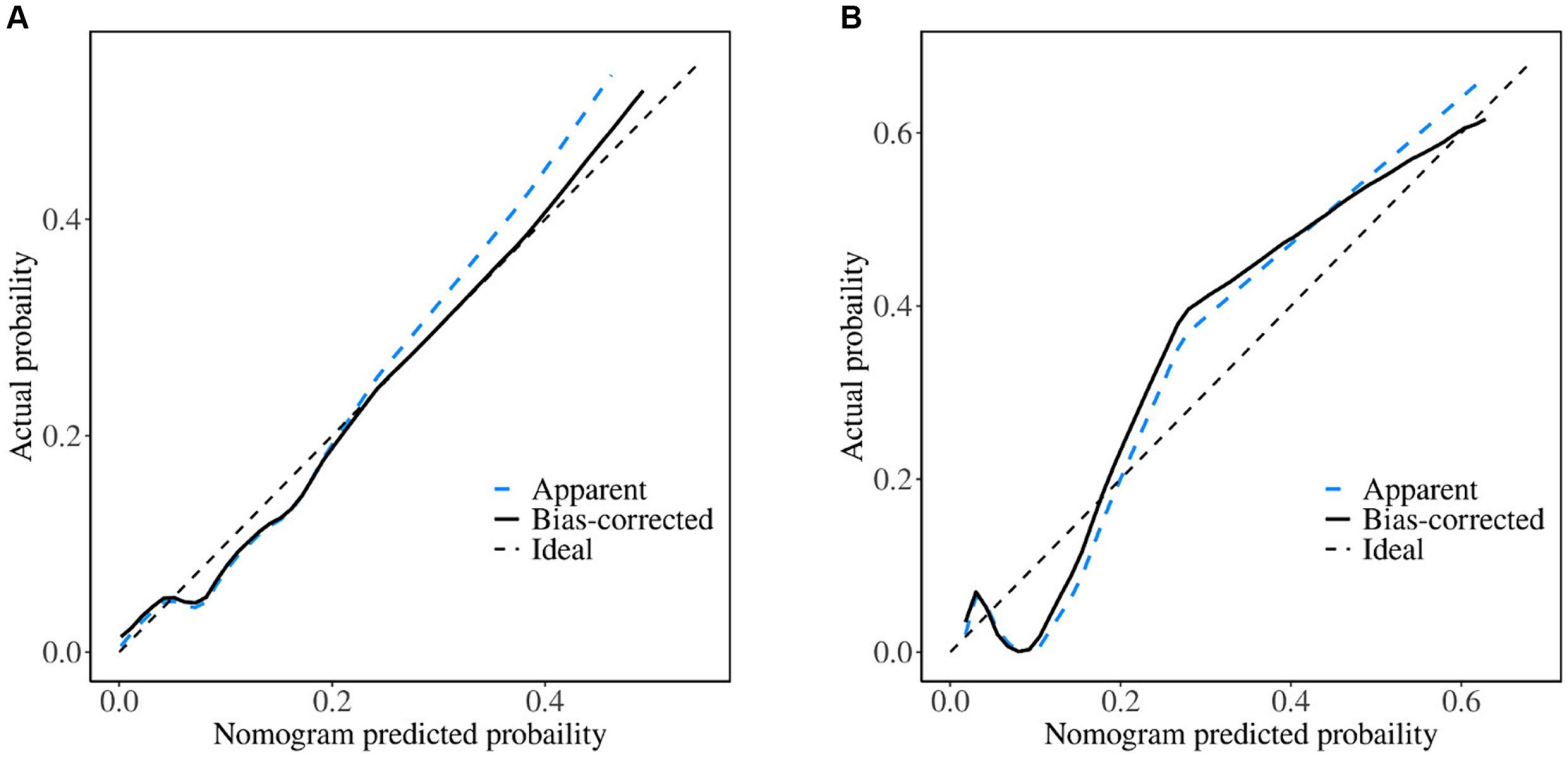
Calibration curves of the nomogram. **(A)** Training set; **(B)** validation set. When the solid line (performance nomogram) is nearer the dashed line (ideal model), the nomogram’s prediction accuracy is higher.

**Figure 6:**
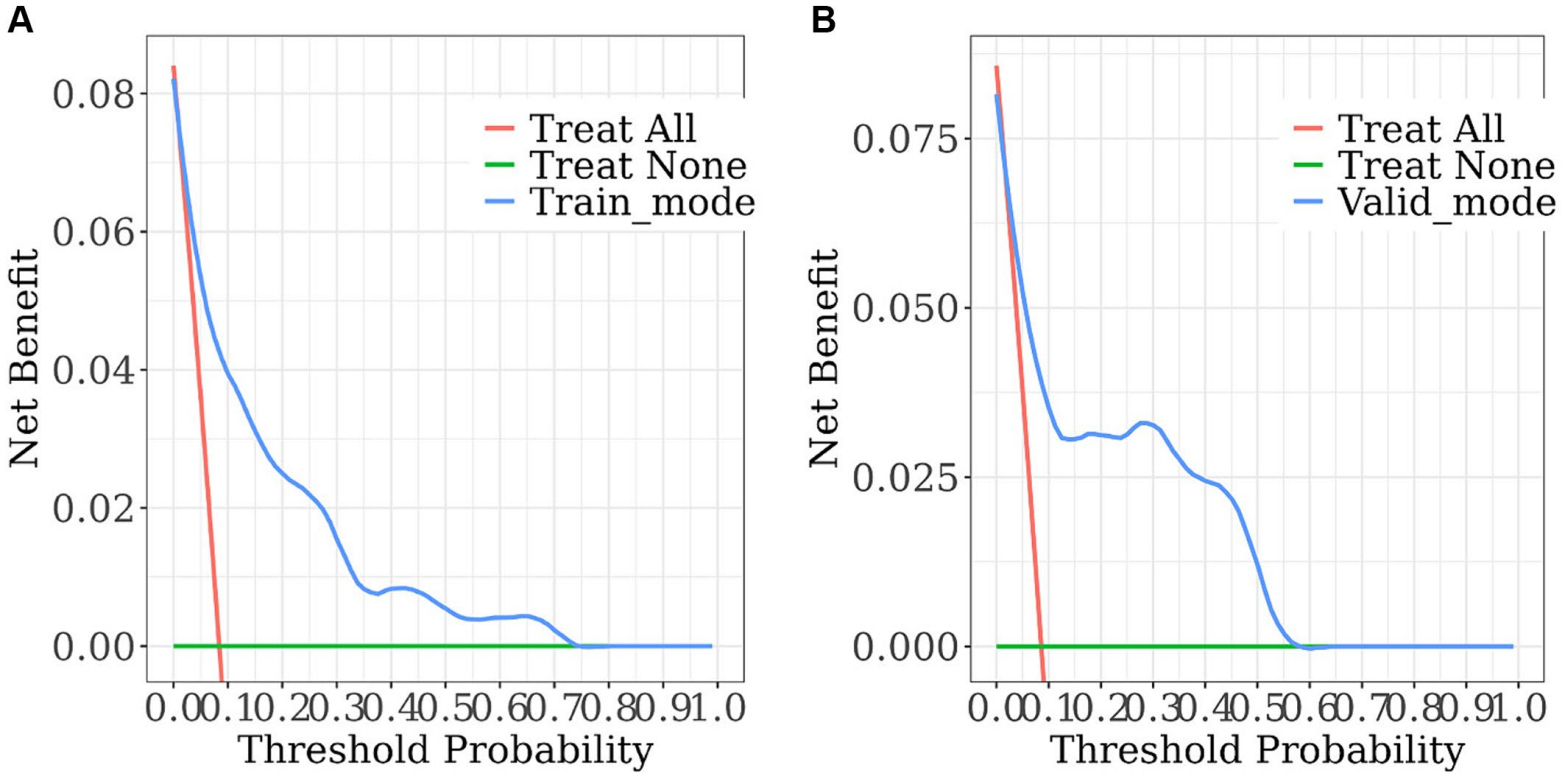
The decision curve analysis of the nomogram. **(A)** Training set; **(B)** validation set. The horizontal solid line denotes patients without SWO, the red line represents all SWO patients, and the solid blue line comes from the prediction model. The estimated net benefit per patient for the nomogram prediction of SWO risk is shown in the figure. As the model curve is stretched, the net benefit rises.

We created a prediction model that took UA, C3, IL-8, FA, FCP, eosinophil, and hypertension into account. The total scores for all the predictor variables were added up, and a vertical line representing the likelihood of SWO was projected down at the total score. Each predictor variable was calculated as a particular score on a grading scale. A higher probability of SWO is indicated by higher scores (Figure 3). For this nomogram, the area under the ROC curve (AUC) was 0.829 (95% CI, 0.760–0.899; Figure 4A). The calibration curves plotted based on 1,000 resamplings of the bootstrap method show a good fit between the actual probabilities in the nomogram and the predicted probabilities, which demonstrates the accuracy and stability of the predictive model (Figure 5A). The curves demonstrated that when the risk threshold was between 0 and 0.75, the model had a net benefit (Figure 6A).

#### 4.2.2 Model validation

The nomogram demonstrated good stability and predictive performance in a 25% randomised internal validation. In the validation cohort, the model’s AUC was 0.835 (95% CI: 0.696, 0.975; Figure 4B). The validation cohort’s nomogram calibration curves further demonstrated the model’s good calibration (Figure 5B). When the risk threshold is between 0 and 0.55, the decision curve demonstrates that the model has a net benefit (Figure 6B).

## 5 Discussion

Logistic regression analyses showed significant associations between SWO and hypertension, IL-8, UA, and C3. We also looked at the groups of subjects who were obese or had severe WHM, and we found that hypertension was a shared risk factor for all three, while the other characteristics were exclusively linked to SWO. We evaluated WMH using the Fazekas scale, a visual rating scale that was frequently employed in clinical settings to score WMH. The study by Zeng et al. (2020) revealed that participants would begin to exhibit significant deterioration in cognitive performance and white matter architecture at a Fazekas score greater than 3, thus we selected a cut-off value of 3 to reflect this. We identified unique risk factors for SWO through the present study and developed two predictive models, including the nomogram plots and the HUCIFE scoring system, to predict the prevalence of SWO. Factors including hypertension, IL-8, UA, C3, and the synergistic variables such as FA, FCP, and eosinophil were adopted in the nomogram plots and the HUCIFFE scoring tables for predicting SWO. The two models had excellent performance with ROC and C indices of 0.829 and 0.828, respectively. These two models only required blood sample testing to operate and did not rely on magnetic resonance imaging. They could be used to screen the prevalence of SWO more conveniently, more economically and earlier, and laid the foundation for timely and further intervention of SWO.

The most significant risk factor for WMH was hypertension (Dufouil et al., 2001; de Leeuw et al., 2002), which was defined as having a systolic blood pressure (SBP) of less than 130 mmHg or a diastolic blood pressure (DBP) of less than 80 mmHg (Whelton et al., 2018). From the early 20th century, there has been recognition of the link between obesity and hypertension (Page, 1969). The general consensus is that the prevalence of obesity is positive correlated with hypertension. Insulin, leptin, the renin-angiotensin-aldosterone system (RAAS), sodium excretion, and stress natriuresis were some of the mechanisms by which obesity raised blood pressure (Landsberg et al., 2013). Mechanisms contributing to white matter lesions in the brain include gliosis, endothelial dysfunction, hypoperfusion and oxidative stress, all of them might be exacerbated by hypertension. It was suggested that BBB damage played a role in the etiology of WMH (Teoh et al., 2009; Wardlaw et al., 2015). This mechanism also encouraged BBB damage when hypertension was present, which worsened neuroinflammation in the brain (Toth et al., 2013). When compared to normotensive people, hypertension patients had a significantly higher relative risk of both subcortical WMH and periventricular WMH (de Leeuw et al., 2002). Hypertension thus played an important role in the pathogenesis of SWO by promoting and exacerbating the process.

Uric acid was a naturally occurring water-soluble antioxidant that efficiently scavenged the majority of reactive nitrogen and oxygen radicals in the peripheral nervous system (Gao et al., 2008) and provided the peripheral nervous system with good defense against oxidative stress (Bowman et al., 2010). Adipose tissue had the ability to make and secrete uric acid (Tsushima et al., 2013), and hyperuricaemia was twice as common in obese people as it was in healthy individuals (Liu et al., 2015). This was further supported by a study on fat mice, who had increased blood uric acid levels (Tsushima et al., 2013). Nitric oxide, according to Vannorsdall et al. (2008), might be mainly responsible for the effects of uric acid on the brain. High uric acid levels might decrease the nitric oxide availability, which would impair vascular tone and endothelial function, resulting in the development of WMH. High levels of uric acid in the CSF could subsequently impair the BBB (Bowman et al., 2010), which aided in the formation of WMH. Even so, some researches indicated that uric acid was not related to WMH (Kim et al., 2020). The opposite results might be explained by the various study subjects. Not all types of WMH were associated with uric acid. There were many aspects in the pathogenesis of WMH, different subtypes might have different pathogenesis. This might be the reason why uric acid was closely associated with some WMH subtypes, such as SWO, but not with the others. Uric acid might play a significant role in the etiology of SWO by reducing nitric oxide availability, harming the blood–brain barrier, and impairing endothelial function and vascular tone.

The most prevalent complement, C3, is a crucial part of innate immunity and is involved in immunological control and infection protection (Frank and Volanakis, 1998). Additionally, C3 levels are greater in obese individuals than in non-obese individuals and are most strongly correlated with abdominal obesity in particular, with this manifestation being more pronounced in extremely obese individuals (Hernández-Mijares et al., 2007). An animal experiment demonstrated that visceral adipocytes from obese mice upregulated the expression of PU.1, a member of the Ets family of DNA-binding proteins which controled the expression of inflammatory factors and enzymes that increase levels of chronic inflammation, such as tumour necrosis factor and interleukins *in vivo* (Lin et al., 2012). These inflammatory factors also stimulated adipocytes to express C3 mRNA, thus raised serum C3 levels. The protease histonectin L (CTSL) cleft C3 into two pieces, C3a and C3b (Asavapanumas et al., 2021), and once cleft, C3a bound to its receptor C3aR, promoting vascular inflammation and BBB damage (Propson et al., 2021). Insufficient perfusion of C3a receptors caused white matter injury in microglia, which was exacerbated by elevated C3 levels (Zhang et al., 2020). This could likewise play a role in the etiology of SWO.

IL8 functioned as a chemokine, inducing chemotaxis in macrophages, neutrophils, basophils, and T cells (Hammond et al., 1995; Bonecchi et al., 2000; Straczkowski et al., 2002; Janeway, 2012; Turner et al., 2014). IL-8 was generated and secreted by human adipocytes and Bruun et al. (2000, 2001) demonstrated that blood IL-8 levels were higher in obese individuals, which could led to increased local and systemic inflammation as well as insulin resistance (Gregor and Hotamisligil, 2011; Lin et al., 2020). This localized and widespread inflammation brought about microvascular alterations and created a chronic underperfusion, which in turn caused persistent oligodendrocyte mortality and ongoing degeneration of myelin fibres, resulting in progressive white matter injury (Sandu et al., 2015). Moreover, IL-8, which increased pro-inflammatory and pro-oxidant nitric oxide and affected the cerebral microvascular endothelium, might be linked to cytokine overexpression associated with microglia activation, resulting in WMH (Sloane et al., 1999; Frodl and Amico, 2014). The pathogenesis of SWO was significantly influenced by the chronic perfusion deficit condition and increased nitric oxide processes brought on by IL-8, which promoted oligodendrocyte death in SWO.

Earlier studies focused more on the predictive value of individual parameters. However, when determining a patient’s diagnosis and prognosis for a disease, doctors naturally combine a number of patient characteristics and symptoms, such as predictors and test results, and probability estimates are rarely based on individual predictors. Prediction is therefore by its very nature multivariate (Collins et al., 2015). Combinations of two or more parameters or raising the parameter of related can be utilized to enhance the diagnostic performance since several risk variables included in a model might produce synergistic effects. Based on the present study, we finally constructed a prediction model that included hypertension, UA, C3, IL-8, FA, FCP and eosinophil. The results of the ROC analysis in this study demonstrate the excellent performance of the model. The model makes it easy for clinicians to predict the probability of obese brain white matter lesions. For each individual patient, we can substitute the patient’s data into a nomogram (Figure 3) and add up the scores obtained for each item, and the probability corresponding to the total score is the patient’s risk of developing the disease. The model is straightforward and understandable, making it simple to use in routine clinical practice. Moreover, we can estimate risk using the HUCIFFE scale (Table 9), where a total score of 9 or higher indicates a high risk for SWO. The two measures can be used to validate one another and mainly used for SWO screening. This technology enables for fast risk assessment, as well as screening at community hospitals, nursing homes, and other locations without access to MRI, allowing for early detection and intervention and preserving vital medical resources. So that we can better control the hazards of WMH.

The present study also has some limitations. The subjects in this study were provided by a single centre and the Chinese obesity criteria (BMI ≥28 kg/m^2^) were used, and the model might limit its generalizability. Even though bootstraps with 1,000 resamples were used to confirm our nomogram, additional prospective multicenter studies were still required to verify our findings externally. The average age of the subjects in this study is older, while older subjects exhibit higher WMH volumes. BMI was adopted as the only marker of obesity and there were no available data on other anthropometric markers of obesity (e.g., waist-hip circumference or waist circumference), which might be biased. In addition, WMH can be divided into PVWMHs and DWMHs, and obesity can be divided into subcutaneous fatty and visceral fatty. This subdivision into specific subtypes could better improve the predictability of SWO. Last but not least, our study’s sample size was somewhat modest and has to be expanded in order to further validate the results.

## 6 Conclusion

According to our study, we used the synergistic variables FA, FCP, and eosinophil for SWO along with hypertension, IL-8, UA, and C3 to create a nomogram (Figure 3) and a HUCIFFE scale (Table 9). The nomogram enables visualization of the patient’s WMH risk. When the HUCIFFE scale is scored higher than 9, the patient has a significantly increased risk of developing the disease. The two forms can be compared and validated against one another, and they can be widely used in clinical practice. These two models only require blood sample testing to operate and do not rely on magnetic resonance imaging. The screening can be conducted in community hospitals, nursing homes, and other locations without access to MRI, allowing for the earliest possible detection and intervention as well as providing assessment criteria for the early identification and control of WMH hazards.

## Data availability statement

The original contributions presented in the study are included in the article/Supplementary material, further inquiries can be directed to the corresponding authors.

## Ethics statement

The studies involving humans were approved by the Ethics Committee of Luwan Branch of Ruijin Hospital, Shanghai Jiao Tong University School of Medicine. The studies were conducted in accordance with the local legislation and institutional requirements. The participants provided their written informed consent to participate in this study.

## Author contributions

FC: Conceptualization, Data curation, Formal analysis, Investigation, Methodology, Project administration, Validation, Visualization, Writing – original draft, Writing – review & editing. L-hC: Formal analysis, Investigation, Methodology, Writing – review & editing. F-yM: Investigation, Writing – review & editing. L-lZ: Conceptualization, Formal analysis, Investigation, Writing – review & editing. J-rH: Conceptualization, Formal analysis, Funding acquisition, Investigation, Methodology, Project administration, Resources, Software, Supervision, Validation, Writing – original draft, Writing – review & editing.
