# Supplementary material for: Development and validation of a predictive model for severe white matter hyperintensity with obesity
Source: Front Aging Neurosci. 2024 Jun 3;16:1404756. doi: 10.3389/fnagi.2024.1404756 (PMC11180876; doi:10.3389/fnagi.2024.1404756)
Supplement: Supplementary file 1 [file Table_1.docx]

Supplementary Table 1 Demographic data and clinical characteristics of the development and validation team

| Variable | Total (n = 650) | Training group (n = 487) | validation group (n = 163) | | P value | |
| --- | --- | --- | --- | --- | --- | --- |
| Age (year) | 66.89 ± 11.84 | 66.64 ± 12.22 | | 67.63 ± 10.63 | | 0.359 |
| Sex（n,%） |  |  | |  | | 0.260 |
| Male | 251 (38.62) | 182 (37.37) | | 69 (42.33) | |  |
| Female | 399 (61.38) | 305 (62.63) | | 94 (57.67) | |  |
| Hypertension | 426 (65.54) | 314 (64.48) | | 112 (68.71) | | 0.325 |
| Diabetes | 203 (31.23) | 145 (29.77) | | 58 (35.58) | | 0.166 |
| Hyperlipidemia | 227 (34.92) | 172 (35.32) | | 55 (33.74) | | 0.715 |
| Cardiac disease | 89 (13.69) | 62 (12.73) | | 27 (16.56) | | 0.218 |
| Previous strokes | 209 (32.15) | 154 (31.62) | | 55 (33.74) | | 0.616 |
| Smoking | 123 (18.92) | 90 (18.48) | | 33 (20.25) | | 0.619 |
| WBC (10^9/L) | 6.39 ± 1.87 | 6.35 ± 1.76 | | 6.51 ± 2.16 | | 0.388 |
| Neutrophil (10^9/L) | 4.02 ± 4.32 | 4.04 ± 4.89 | | 3.95 ± 1.79 | | 0.818 |
| Lymphocyte(10^9/L) | 1.91 ± 0.63 | 1.91 ± 0.63 | | 1.93 ± 0.65 | | 0.631 |
| Monocyte (10^9/L) | 0.43 ± 0.16 | 0.42 ± 0.15 | | 0.44 ± 0.19 | | 0.398 |
| Basophil (10^9/L) | 0.34 ± 0.22 | 0.34 ± 0.22 | | 0.34 ± 0.22 | | 0.645 |
| Eosinophil (10^9/L) | 0.18 ± 0.21 | 0.18 ± 0.20 | | 0.18 ± 0.22 | | 0.971 |
| ALT (IU/L) | 21.74 ± 16.39 | 21.74 ± 16.07 | | 21.74 ± 17.37 | | 0.998 |
| AST (IU/L) | 22.30 ± 14.35 | 21.85 ± 9.49 | | 23.63 ± 23.51 | | 0.171 |
| Albumin (g/L) | 38.34 ± 3.34 | 38.45 ± 3.34 | | 38.00 ± 3.33 | | 0.133 |
| BUN (mmol/L) | 5.76 ± 1.94 | 5.68 ± 1.88 | | 5.99 ± 2.11 | | 0.077 |
| Cr (umol/L) | 73.74 ± 21.95 | 73.55 ± 22.10 | | 74.34 ± 21.56 | | 0.691 |
| UA (umol/L) | 346.24 ± 96.66 | 346.58 ± 95.64 | | 345.20 ± 99.93 | | 0.875 |
| Complement (U/mL) | 53.23 ± 12.39 | 52.96 ± 12.24 | | 54.04 ± 12.83 | | 0.337 |
| TC (mmol/L) | 4.56 ± 1.16 | 4.53 ± 1.16 | | 4.64 ± 1.16 | | 0.309 |
| TG (mmol/L) | 1.55 ± 1.03 | 1.56 ± 1.07 | | 1.51 ± 0.91 | | 0.582 |
| HDL (mmol/L) | 1.12 ± 0.38 | 1.12 ± 0.41 | | 1.12 ± 0.29 | | 0.988 |
| LDL (mmol/L) | 2.88 ± 0.87 | 2.86 ± 0.87 | | 2.93 ± 0.88 | | 0.366 |
| Free IL-2 (pg/mL) | 535.93 ± 254.26 | 523.63 ± 224.02 | | 572.83 ± 326.82 | | 0.085 |
| IL-β (pg/mL) | 3.82 ± 8.35 | 4.06 ± 9.08 | | 3.10 ± 5.61 | | 0.220 |
| TNF-α (pg/mL) | 24.24 ± 23.60 | 24.08 ± 25.53 | | 24.72 ± 16.55 | | 0.771 |
| IL-10 (pg/mL) | 1.64 ± 5.26 | 1.71 ± 5.99 | | 1.40 ± 1.69 | | 0.528 |
| IL-8 (pg/mL) | 161.52 ± 254.03 | 165.18 ± 278.83 | | 150.55 ± 158.04 | | 0.538 |
| IL-6 (pg/mL) | 7.68 ± 39.25 | 8.59 ± 45.20 | | 4.93 ± 4.81 | | 0.321 |
| FA (ng/mL) | 9.61 ± 5.34 | 9.60 ± 5.33 | | 9.67 ± 5.37 | | 0.880 |
| VitB12 (pg/mL) | 400.08 ± 299.10 | 383.78 ± 277.13 | | 448.69 ± 353.18 | | 0.034 |
| HbA1c (%) | 6.39 ± 1.36 | 6.38 ± 1.37 | | 6.43 ± 1.32 | | 0.674 |
| IgA (mg/dL) | 264.40 ± 113.37 | 266.41 ± 114.77 | | 258.38 ± 109.20 | | 0.438 |
| IgG (mg/dL) | 1193.89 ± 302.08 | 1191.71 ± 313.61 | | 1200.45 ± 265.20 | | 0.751 |
| IgM (mg/dL) | 95.83 ± 61.63 | 97.58 ± 62.87 | | 90.56 ± 57.60 | | 0.213 |
| CRP (mg/dL) | 0.63 ± 1.55 | 0.60 ± 1.53 | | 0.71 ± 1.61 | | 0.467 |
| C3 (mg/dL) | 91.25 ± 17.20 | 91.12 ± 17.35 | | 91.66 ± 16.77 | | 0.728 |
| C4 (mg/dL) | 22.48 ± 6.57 | 22.25 ± 6.39 | | 23.17 ± 7.05 | | 0.126 |
| 25OH-VitD(nmol/L) | 49.60 ± 22.02 | 49.71 ± 21.24 | | 49.27 ± 24.26 | | 0.835 |
| FBG (mg/dL) | 5.81 ± 1.81 | 5.85 ± 1.86 | | 5.67 ± 1.66 | | 0.274 |
| 2hPBG (mg/dL) | 9.75 ± 3.47 | 9.76 ± 3.57 | | 9.73 ± 3.16 | | 0.948 |
| FCP (mg/mL) | 2.72 ± 1.23 | 2.64 ± 1.21 | | 2.95 ± 1.27 | | 0.005 |
| 2hPCP (ng/mL) | 9.45 ± 6.89 | 9.49 ± 7.30 | | 9.35 ± 5.49 | | 0.866 |
